# Does an electronic cognitive aid have an effect on the management of severe gynaecological TURP syndrome? A prospective, randomised simulation study

**DOI:** 10.1186/s12871-017-0365-8

**Published:** 2017-05-30

**Authors:** Michael St.Pierre, Georg Breuer, Dieter Strembski, Christopher Schmitt, Bjoern Luetcke

**Affiliations:** Anästhesiologische Klinik, Universitätsklinikum Erlangen, Krankenhaustrasse 12, 91054 Erlangen, Germany

**Keywords:** Acute hyponatraemia, Checklist, Guidelines, Simulation, TURP syndrome

## Abstract

**Background:**

Lack of familiarity with the content of current guidelines is a major factor associated with non-compliance by clinicians. It is conceivable that cognitive aids with regularly updated medical content can guide clinicians’ task performance by evidence-based practices, even if they are unfamiliar with the actual guideline. Acute hyponatraemia as a consequence of TURP syndrome is a rare intraoperative event, and current practice guidelines have changed from slow correction to rapid correction of serum sodium levels. The primary objective of this study was to compare the management of a simulated severe gynaecological transurethral resection of the prostate (TURP) syndrome under spinal anaesthesia with either: an electronic cognitive aid, or with management from memory alone. The secondary objective was to assess the clinical relevance and participant perception of the usefulness of the cognitive aid.

**Methods:**

Anaesthetic teams were allocated to control (no cognitive aid; *n* = 10) or intervention (cognitive aid provided; *n* = 10) groups. We identified eight evidence-based management tasks for severe TURP syndrome from current guidelines and subdivided them into acute heart failure (AHF)/pulmonary oedema tasks (5) and acute hyponatraemia tasks (3). Implementation of the treatment steps was measured by scoring task items in a binary fashion (yes/no). To assess whether or not the cognitive aid had prompted a treatment step, participants from the cognitive aid group were questioned during debriefing on every single treatment step. At the end of the simulation, session participants were asked to complete a survey.

**Results:**

Teams in the cognitive aid group considered evidence-based treatment steps significantly more often than teams of the control group (96% vs. 50% for ‘AHF/pulmonary oedema’ *p* < 0.001; 79% vs. 12% for ‘acute hyponatraemia’ *p* < 0.001). Without the cognitive aid, performance would have been comparable across both groups. Nurses, trainees, and consultants derived equal benefit from the cognitive aid.

**Conclusions:**

The cognitive aid improved the implementation of evidence-based practices in a simulated intraoperative scenario. Cognitive aids with current medical content could help to close the translational gap between guideline publication and implementation in acute patient care. It is important that the cognitive aid should be familiar, in a format that has been used in practice and training.

**Electronic supplementary material:**

The online version of this article (doi:10.1186/s12871-017-0365-8) contains supplementary material, which is available to authorized users.

## Background

Specialist societies commonly produce clinical practice guidelines that are based on reviews of the literature, in order to assist clinicians in providing high-quality, evidence based care [1]. Unfortunately, it seems that these clinical practice guidelines are not always implemented by clinicians at the bedside, during the provision of care [2, 3]. There are complex reasons behind the hesitant uptake of guidelines by physicians, but a lack of familiarity with the content of specific guidelines is among the most salient factors associated with non-compliance [1, 4, 5]. Additionally, even if clinicians are familiar with the current content of a guideline, the stress of an emergency may alter an individual’s cognitive functions (e.g., attention, working memory, long-term memory), and analysis-driven decision-making. As a result, omissions of critical steps, practice variability, and non-compliance to established guidelines can increase [2].

Crisis-related cognitive aids (CAs) such as ‘emergency manuals’ [6], ‘emergency quick reference guides’ [7, 8], and ‘crisis checklists’ [9], have been developed for a variety of intraoperative emergencies, to assist clinicians in executing the complex decision making involved in diagnosis and therapy. Ideally, they contain current medical content and provide localised information (e.g., important phone numbers, storage for rarely used drugs) that may help increase the speed and fluidity of performance [10–12].

Several CAs have been tested in simulation studies [10, 13]. Overall, successful adherence to lifesaving procedures was more common among teams using crisis checklists compared to teams without access to CAs [10, 14–16]. In the majority of these studies, participants had been familiarised with the medical content of the CA prior to the study scenario. However, it is conceivable that clinicians’ task performance in rare emergencies can be guided by evidence-based practices even if they are unfamiliar with the actual guidelines. As a result, the use of CAs with current medical content could help to close a translational gap in acute patient care.

We chose a simulated case of severe gynaecological TURP syndrome as a model to test this assumption for two reasons. First, the recently revised “Clinical practice guideline on diagnosis and treatment of hyponatraemia” [17] published by the Hyponatraemia Guideline Development Group of the European Society of Endocrinology, the European Society of Intensive Care Medicine, the European Renal Association, and the European Dialysis and Transplant Association has changed the first-hour management recommendations of acute hyponatraemia from slow to rapid correction of plasma sodium concentration. Second, severe TURP syndrome is a rare intraoperative emergency with a complex cardiopulmonary, haematological, and neurological pathophysiology.

We tested the hypothesis that the use of a CA on TURP syndrome would: a) prompt clinicians to a rapid correction of acute hyponatraemia, and b) improve consideration of recommended tasks for acute heart failure (AHF)/pulmonary oedema.

The primary objective of the present study was to compare the management of severe gynaecological TURP syndrome with either an electronic CA, or with management from memory alone. The secondary objective was to determine the perception of participants of the usefulness and clinical relevance of the CA.

## Methods

### Participants

**Fig. 1:**
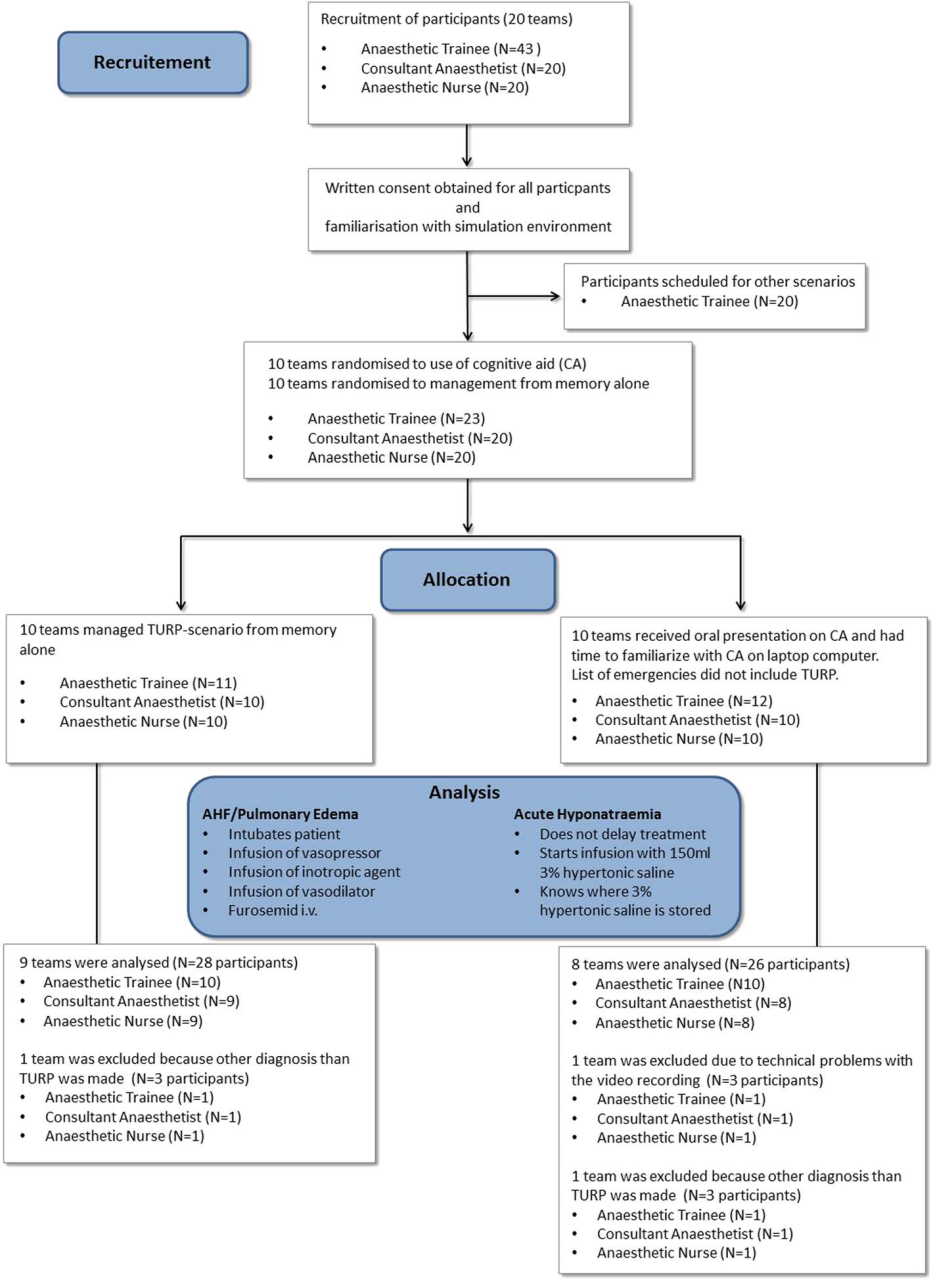
CONSORT flow chart of recruitment, randomisation, and analysis. CA; cognitive aid

After obtaining approval of the study protocol by the ethics committee of the Friedrich-Alexander University Erlangen-Nuremberg (reference number 270_15), we enrolled 83 participants into this prospective, randomised single-blinded controlled trial (Fig. 1). Participants were part of a 20-day institutional training program at the authors’ department. Scheduling for each day reflected the actual role composition of anaesthetic teams commonly found in German anaesthesia departments. Teams consisted of one or two anaesthetic trainees, a consultant anaesthetist, and an anaesthetic nurse. Written informed consent was obtained from all participants prior to the second scenario. Participants from both groups were briefed on the scenario with a standard amount of information. The script for the intervention group was supplemented with a final passage reminding participants to use the CA once a diagnosis had been made.

### Study protocol

The first two scenarios of the day (i.e., severe gynaecological TURP syndrome, cardiac emergency during C-section under spinal anaesthesia [18]) were randomly alternated using a web-based tool (www.randomizer.org). All participants managed their first scenario from memory alone (control group). Following standardised educational intervention participants were able to access the CA during the second scenario (cognitive aid (CA) group). Standardised educational intervention consisted of a didactic and practical training. The aim of the didactic training was to familiarise participants with the concept of CAs and with the alternative approaches of using them during an emergency (i.e. in a prospective “challenge-response” manner or in a retrospective “do-verify” manner). During practical training, participants were given the opportunity to familiarise themselves with the structure and content of the CA by trying it out on a laptop computer. In this training version, the list of emergencies did not include the emergency to follow in the next scenario. The study protocol required that every scenario where participants made diagnoses other than TURP syndrome had to be excluded from further analysis.

### Cognitive aid

**Fig. 2:**
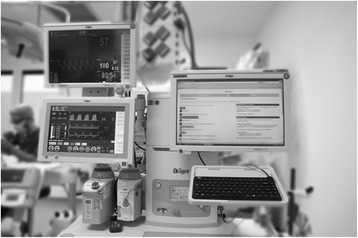
Location of anaesthesia information management system with web-based cognitive aid. Placement provides easy accessibility and consistent location, without interfering with routine work flow. The layout was adapted from a paper based template available from Ariadne Labs [9]

The CA was available as a set of HTML5-pages throughout the simulation and accessible via a web-browser on the institution’s anaesthesia information management system mounted on the anaesthesia machine (Fig. 2). Each HTML5-page started with a header containing the identification and description of the emergency, followed by a bold type statement regarding the management priority. Capitalising on the monitor’s landscape format, we chose a two-columned layout with action steps on the left and reference information on the right side, as done previously [9, 19, 20]. The design of the CA followed recommendations for CAs in medicine [21], as well as design guidance for electronic emergency checklists in aviation [22, 23]. A prototype was developed and tested by the authors before its first use during the study trial. The medical content of the CA on “Intraoperative TURP syndrome” was developed by reviewing guidelines from recognised bodies on acute hyponatraemia [17, 24] and on AHF [25], as well as commercial [26, 27] and open access emergency manuals [28, 29]. Following an iterative process, the final action items for the checklist were selected by consensus among the authors and adapted to local conditions. An additional file shows the translated text of the original German version of the CA on “TURP syndrome” (see Additional file 1). The CA used during the simulation contained a list with 36 adult and paediatric emergencies. This was to ensure that participants selected the diagnosis from clinical cues, and not out of convenience due to only having one scenario available.

### Scenario

The crisis scenario was based on published case reports of severe hyponatraemia and fluid overload during hysteroscopic surgery [30–33]. A middle-aged woman underwent hysteroscopic resection of multiple uterine myomas. At her request, the procedure was performed under spinal anaesthesia. In the scenario, anaesthetic care was handed off to the participants by the primary anaesthetist, played by a member of the simulation team. At that time the operation was 1.5 h into the dissection of the submucosal myoma. Unforeseen problems with endometrial ablation were a reason for the prolonged surgical time. After an initial regular conversation with the anaesthetist, the patient started to complain about nausea, headache, and shortness of breath. The symptoms the patient complained about were typical for TURP syndrome in an awake patient, to increase the probability that participants’ would make the correct diagnosis. Symptoms worsened over 5 to 10 min with a concomitant drop in saturation and a gradual rise in blood pressure. The patient became confused, seized, and was no longer responsive. Saturation dropped (target SpO_2_: 85%), and the patient became hypotensive (target blood pressure: 80/45 mmHg). Auscultation revealed wet rales. The first blood gas and electrolyte analysis revealed metabolic acidosis (pH 7.28), low plasma sodium chloride concentration (Na+: 104 mmol/l; Cl- 80 mmol/l), and anaemia (Hb 8 g/dl).

Haemodynamic and pulmonary variables were programmed into a manikin-based simulator (SimMan; Laerdal Norway). The protocol was pretested before study commencement using nonparticipating subjects, with the aim of improving clinical cues for the participants. The results served to refine the programming and simulation script.

During the scenario, three members of the simulation team played the roles of gynaecologists and circulating nurses. They did not assist the participants in implementing the critical steps. However, one of the gynaecologists repeatedly complained about spilling of irrigation fluid onto the drapes and the floor. Additionally, three empty bags (10 l) of an electrolyte-free and hypoosmotic distension medium (Purisole® SM) hung clearly visible to participants on an infusion pole.

The consultant anaesthetist was sequestered in a separate room, and could be summoned for help by the primary anaesthetist if requested.

### Assessment tools and scoring

Team performance was assessed using a scenario-specific 8-item checklist. The items were generated by identifying eight evidence-based metrics of essential care for severe TURP syndrome from current guidelines, and by subdividing them by consensus into tasks addressing AHF/pulmonary oedema (five tasks [25]), and tasks addressing acute hyponatraemia (three tasks [17]). Two additional clinically relevant tasks were added by consensus by the authors (e.g., “calls for help early”, “Increases PEEP”; Table 1). We did not develop a scoring system by assigning points for the various treatment steps as others have done [15]. Instead, we confined ourselves to scoring task items in a binary fashion (yes/no) and to weighting them equally. We recorded how often the monitor with the CA was accessed during the scenario either by an individual or by the entire team. We did not measure any time frame within which key processes had to be performed, as reported by other research teams [20]. In the intervention group, we attributed an action or a consideration of the use of the CA if: a) the anaesthetist read aloud the treatment step and then either gave the order or started to discuss the measure with team members or the gynaecologist, or b) an individual’s action immediately followed reading the CA, or c) the trainee or consultant stated during debriefing that the action or consideration had been in response to an item of the CA. As key processes were binary outcomes (yes/no), we assumed that adjudications of actions could be easily made.

**Table 1:** Specific task performance and task consideration data

	Class and level^b,c^	Cognitive Aid (*n* = 8)	No Cognitive Aid (*n* = 9)	Differences in adherence
Acute Heart Failure and Pulmonary Oedema				
• Calls for help early	n/a	8 (100.0)	9 (100.0)	0%^a^
• Intubates patient	IC	8 (100.0)	9 (100.0)	0%^a^
• Increases PEEP above respirators’ default value of 5 mbar	n/a	7 (87.5)	3 (33.0)	54.5%
• Considers or starts infusion of vasopressor	IIb B	7 (87.5)	2 (22.0)	65.5%
• Considers or starts infusion of inotropic agent	IIb C	8 (100.0)	1 (11.0)	89%
• Considers or starts infusion of IV vasodilator	IIa B	8 (100)	2 (22.0)	78%
• Gives furosemide IV	IC	8 (100)	6 (67.0)	33%
Acute Hyponatraemia				
• Does not delay treatment of hyponatraemia but initiates prompt infusion of hypertonic saline	1D	4 (50.0)	1 (11.0)	39%
• Starts infusion with recommended dose of 150 ml 3% hypertonic saline	1D	8 (100.0)	1 (11.0)	89%
• Knows that pre-prepared 150 ml bottles of 3% hypertonic saline are stored at the ICU	1D	7 (87.5)	1 (11.0)	76.5%

*AHF* acute heart failure, *PEEP* positive endexpiratory pressure, *SBP* systolic blood pressure

Values in column 3 (Cognitive Aid) and 4 (No Cognitive Aid) are number of teams correctly considering or performing the task. Values in column 5 are differences in adherence to the individual task between the cognitive aid group and the control group

^a^With the exception of the tasks “Calls for help early” and “Intubates patient” the adherence for tasks was higher in the cognitive aid group

^b^Class of recommendation and level of evidence as stated in the ESC Guidelines for the diagnosis and treatment of acute and chronic heart failure [25]

^c^Strength of recommendation and quality of evidence (GRADE Methodology) as stated in the Clinical practice guideline on diagnosis and treatment of hyponatraemia [17]

n/a = not applicable

A list of six survey items regarding the usefulness and clinical relevance of CAs was generated from a literature review by one of the authors (MS). Face validity and content validity were assessed via discussion by the five authors. Responses to survey questions were binary (agree/don’t agree) (Table 2). Because all anaesthetists and anaesthetic nurses were candidates of the institutional training program, we were unable to pilot test the survey on a subpopulation within our department prior to the study.

**Table 2:** Survey questions regarding usefulness and clinical relevance of CA (translated from German)

	Trainee (*n* = 10)	Consultant (*n* = 8)	Nurse (*n* = 8)
I found the CA helpful because it reminded me of treatment steps I otherwise might have forgotten.	5 (50)	5 (62.5)	4 (50)
I found the CA helpful because we could check our treatment steps for completeness.	5 (50)	5 (62.5)	5 (62.5)
I found the CA helpful because it promoted team discussion of our treatment steps.	6 (60)	5 (62.5)	3 (37.5)
I would appreciate the introduction of the CA into daily practice	6 (60)	4 (50)	4 (50)
I would not use the CA in an intraoperative emergency, but I could imagine that inexperienced colleagues may benefit from using it.	0 (0)	0 (0)	0 (0)
For successful implementation of the CA, it would be necessary to establish a ‘code reader’ who would guide the team through all treatment steps.	4 (40)	4 (50)	2 (25)

Items were scored in a binary fashion (agree/don’t agree)

Values are number of participants (%)

*CA* cognitive aid

### Data collection

Multiscreen synchronised video recordings were taken of all 20 scenarios and were available for offline evaluation. To determine whether or not the CA had prompted an action, we interviewed participants from the intervention group during debriefing on every single treatment step. We asked them whether they would have performed the task in any case or whether reading the CA had reminded them of the measure. In addition, we wanted to know whether they had been aware that recommendations concerning the speed of infusion of hypertonic saline had changed in past years, and whether they knew where 3% hypertonic saline was stored in our department.

At the end of the simulation session participants were asked to complete the survey. We explained to the participants that the term ‘code reader’ (question 6) denotes a dedicated person who assists the team leader by reading critical steps aloud from the CA and then acknowledging completion of each step [15].

### Evaluators

Task performance data of all scenarios was evaluated by one of the five authors. Video data of all scenarios were reviewed and scored by a single study team member (MS). Nine random scenarios were independently reviewed by a second team member (BL).

### Statistics

Data were analysed with the use of SPSS software version 21.0 (IBM). All reported *p* values are two-sided, and *p*-values of less than 0.05 were considered statistically significant. Participant characteristics and time intervals were compared with a two-tailed t-test. Task performance data (yes/no) were compared with Fisher’s exact tests, responses to survey questions (yes/no) were analysed by applying the chi-squared test if applicable. Interrater agreement on the scoring of mandatory and optional task completion in nine random scenarios was assessed using Cohens’ kappa statistic.

### Cognitive errors analysis

If participants made diagnoses other than TURP syndrome and then failed to adjust their initial diagnosis in the further course we addressed this issue during debriefing. We interviewed the teams about their decision-making process and related their responses to the most frequent cognitive errors.

## Results

Twenty teams (23 anaesthetic trainees, 20 consultants, and 20 anaesthetic nurses) participated in the study. Technical problems caused the loss of one video recording from the CA group. One team from the CA group and one team from the control group attributed seizures to “Local Anaesthetic Systemic Toxicity” (LAST) and started lipid resuscitation. In both of these teams, no critical steps for severe acute hyponatraemia were performed so that the two scenarios were excluded from further analysis. As a result, data from 17 teams (17 anaesthetic nurses, 20 anaesthetic trainees, and 17 consultants) were analysed (Fig. 1). There were no group differences in terms of years of clinical experience (Table 3). The inter-rater reliability for the scoring of task completion and task consideration showed good agreement (κ = 0.81). On the assumption that that key tasks had binary outcomes (yes/no), consensus between raters was easily achieved for any instance of initial disagreement.

**Table 3:** Participant characteristics: Years of clinical experience

Characteristics	Cognitive Aid		*P* value
	No	Yes	
Consultant	10.7 (±1.3) yrs. (*n* = 9)	12.5 (±2.7) yrs. (*n* = 8)	0.56
Anaesthetist Trainee	3.2 (±0.6) yrs. (*n* = 10)	3.3 (±0.9) yrs. (*n* = 10)	0.9
Anaesthetic Nurse/Assistant	9.7 (±2.8) yrs. (*n* = 9)	10.7 (±3.1) yrs. (*n* = 8)	0.79

Values are mean (SD)

**Fig. 3:**
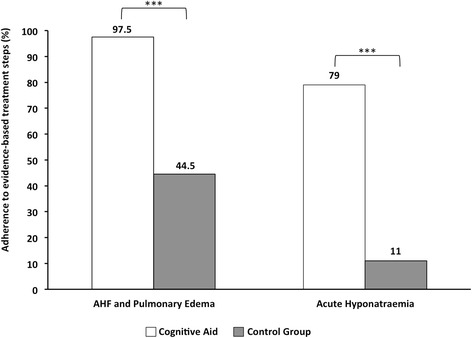
Adherence to critical treatment steps of acute heart failure (AHF) and pulmonary oedema as defined by the guidelines of the European Society of Cardiology [25] and of acute hyponatraemia as defined by the guidelines of the Hyponatraemia Guidelines Development Group [17]. Data on frequency are taken from Table 1: AHF/pulmonary oedema (five evidence-based items) and acute hyponatraemia (three evidence-based items) management tasks. ***p < 0.001

The availability of the CA improved performance in the five treatment tasks of AHF/pulmonary oedema as defined by the guidelines of the European Society of Cardiology [25] [39/40 (97.5%) vs. 20/45 (44.5%) tasks; *p* < 0.001] as well as in the treatment tasks of acute hyponatraemia as defined by the guidelines of the Hyponatraemia Guidelines Development Group [17] [19/24 (79%) vs. 3/27 (11%); *p* < 0.001] (Fig. 3). Teams in the CA group started an infusion of 150 ml 3% saline and knew where to find pre-prepared solutions significantly more often than participants of the control group. With the exception of the tasks “Calls for help early” and “Intubates patient” the adherence for individual tasks was higher in the CA group (33% to 89% difference; Table 1). The data collected during debriefing indicates that the observed difference can be attributed to the use of the CA, as performance from memory alone would have been comparable across both groups (Table 4). Only one of 17 teams (6%) had been aware of the fact that current guidelines recommend prompt infusion of hypertonic saline.

**Table 4:** Without the cognitive aid both groups would have performed equally

	Cognitive Aid (*n* = 8) Debriefing	No Cognitive Aid (*n* = 9) Scenario	*p*-value
	From memory alone	From memory alone	
Acute Heart Failure and Pulmonary Oedema			
• Calls for help early	8 (100.0)	9 (100.0)	1.0
• Intubates patient	8 (100.0)	9 (100.0)	1.0
• Increases PEEP above respirators’ default value of 5 mbar	4 (50)	3 (33)	0.63
• Considers or starts infusion of vasopressor	4 (50)	2 (22)	0.33
• Considers or starts infusion of inotropic agent	3 (37.5)	1 (11.0)	0.29
• Considers or starts infusion of IV vasodilator	3 (37.5)	2 (22)	0.62
• Gives furosemide IV	5 (62.5)	6 (67)	1.0
Acute Hyponatraemia			
• Does not delay treatment of hyponatraemia but initiates prompt infusion of hypertonic saline	0 (0)	1 (11)	1.0
• Starts infusion with recommended dose of 150 ml 3% hypertonic saline	3 (37.5)	1(11)	0.29
• Knows that pre-prepared 150 ml bottles of 3% hypertonic saline are stored at the IC	2 (25.5)	1 (11)	0.57

Data in column 2 (Cognitive Aid) were collected during debriefing, where participants declared whether a task had been performed or considered from memory alone or in response to an item of the cognitive aid. Frequencies in column 3 (No Cognitive Aid) describe task performance during the scenario

Values are number of teams (%)

*CA*, cognitive aid

In seven out of eight scenarios (87.5%), the physician initiated the use of the CA, whereas only once the anaesthetic nurse prompted the team to open the CA (12.5%). In four out of eight scenarios (50%) the CA was used in challenge response mode, while four out of eight teams (50%) used the CA to review the tasks after execution. In our study, we did not find an association between TURP treatment performance and the frequency with which teams accessed the CA.

Participant survey responses pertaining to the simulation experience with the CA, and to the usefulness and clinical relevance of the CA are detailed in Table 2. Trainees, consultants, and anaesthetic nurses found the CA equally helpful. Only a minority of participants voted for the implementation of a designated ‘code reader’.

## Discussion

Our primary finding suggests that an electronic CA can improve implementation of recommended tasks for acute heart failure (AHF)/pulmonary oedema, and can prompt clinicians to a rapid correction of acute hyponatraemia in a simulated case of severe gynaecological TURP syndrome.

In our study, we chose pulmonary and haemodynamic target values that would allow participants to initiate the full range of treatment interventions for AHF as recommended by the task force of the European Society of Cardiology [25]: intubation, intravenous loop diuretics, and starting infusion of vasopressor, inotropic agent, and vasodilators. We expected that participants would treat the patient in accordance with the recommendations, as AHF and pulmonary oedema are among the more common perioperative emergencies. This was not the case in the control group. During debriefing, participants’ responses from the CA group revealed that their performance from memory alone would equally have led to a guideline adherence of less than 50% (Table 4). The observed non-compliance rate is in accordance with a mean guideline adherence rate of 50%, reported in the literature [1, 34]. In the presence of a CA the task consideration and performance rate increased to 97.5% (Fig. 3).

In contrast to cardiopulmonary emergencies, acute hyponatraemia is a rare intraoperative event. Until recently, current best practice advocated the slow correction of serum sodium levels for fear of osmotic demyelinisation syndrome (ODS, i.e., central pontine myelinolysis). Current clinical practice guidelines, however, advocate rapid correction of acute hyponatraemia, as the shortening of the hyponatraemic time span seems to decrease the risk of cerebral oedema and ODS [17, 24]. In our study, only one of seventeen teams (6%) knew that guidelines had recently changed and that they currently recommend prompt infusion of hypertonic saline. From memory alone, all participants from the CA group would have delayed treatment until arrival at the intensive care unit (ICU) (Table 4). Our findings underscore the general claim that a lack of familiarity with the content of specific guidelines is one of the leading causes for non-compliance with said guidelines.

As participants knew from the CA that 3% hypertonic saline (250 ml) was the recommended infusion, all eight teams decided to start infusion with the recommended dose. However, only four out of eight teams contacted ICU and urgently requested infusions for the OR, whereas four teams decided to postpone starting the infusion until they had arrived on ICU. During debriefing, teams of the CA group that did not correct hyponatraemia rapidly declared that they had not noticed the statement on the website that ‘acute (symptomatic) hyponatraemia should be rapidly corrected’ (see Additional file 1). Video analysis revealed that in all instances the person reading the CA skipped the line with the management priority and started with the action items. We explain this observation by the fact that participants were still unfamiliar with the general structure of the CA, despite the educational intervention prior to the scenario. The general structure of every HTML5-page included a bold type statement specifying the management priority prior to the action items (see Additional file 1). Similar cases of perceptual blindness to the presence or the content of unfamiliar CAs have been reported by other research teams (e.g. inability to notice large-format ACLS cognitive aids partially blocking the defibrillator screen [13]). Therefore, repetitive training may improve the familiarity with a CA and enhance the chance of it being used effectively during actual emergencies [10, 11, 13].

CAs not only should contain current medical information, but should provide context-specific information as well [13]. In our scenario, the CA could play to one of its strengths by providing anaesthetists with the information on where to find 3% hypertonic saline infusion. From memory alone, only three out of seventeen teams (17%) would have known this piece of information.

In their responses to the survey questions, anaesthetic nurses, anaesthetic trainees, and consultants equally valued the fact that they had been able to access the CA and to review their treatment steps (Table 2). Several studies reported on participants’ comments that they did not need a CA to manage the emergency effectively [15, 35, 36]. In contrast, none of our participants, and in particular no consultant, stated during debriefing that they preferred to manage an intraoperative emergency without the help of a CA.

We chose acute hyponatraemia as a model to test the assumption that clinicians’ task performance in rare emergencies can be guided by evidence-based practices even if they are unfamiliar with the actual guidelines. However, we are convinced that the results of this study can be generalised beyond severe gynaecological TURP syndrome and that timely actualisation of CAs to current guidelines will help to close a translational gap in acute patient care.

There are a number of study limitations. Given the fact that the study was conducted during the annual 20 days institutional training program we did not perform an a priori sample size calculation. Rather, we used a convenience sample, targeting all participating consultants, anaesthetic trainees, and anaesthetic nurses. As a result, the number of participants leaves the study underpowered to detect a difference for each individual task listed in Table 1. Instead, we compared task adherence on the level of AHF/pulmonary oedema tasks and acute hyponatraemia tasks. Furthermore, we were unable to blind participants as well as video reviewers to the CA use. As a result, there is a risk that the video review and the scoring of tasks could be biased. However, because specific task performance and task consideration data had binary outcomes we are of the opinion that there was only limited room for personal interpretation. Additionally, although we tried to provide as many salient clinical cues as possible to optimise the chance that participants would make the correct diagnosis, two teams did not diagnose TURP syndrome. Rather, they perceptually locked on two salient features of the scenario (i.e. the use of a local anaesthetic for spinal anaesthesia, seizure), committed themselves to a diagnosis of LAST, and then did not adjust their initial diagnosis in the light of later and contradicting information (i.e. hypoxia, pulmonary oedema, wet rales, hyponatraemia, hemodilution). Similar cognitive errors have been observed in scenarios where teams treated a STEMI during caesarean section as amniotic fluid embolism [18] or attributed all symptoms of malignant hyperthermia to hyperthyroidism [15].

As participants were familiarised with the CAs immediately prior to the simulation it is conceivable that we introduced confirmation bias in favour of the use and importance of CAs in practice. This bias may have increased the utilization in the scenario as compared to real perioperative emergencies and may have affected responses in the survey.

Another limitation of the study is the fact that both the CA used in the study, as well as the scoring system for the primary outcome were generated from the same material. Although action items were selected in an iterative process by the authors, it is possible that the scoring system might be scoring adherence to itself, thereby creating an inherent bias.

The comparison of tasks performed from memory alone is based on information participants from the CA group gave during debriefing. This approach inevitably carries the risk that the respondents from the CA group might have made biased statements. As an alternative, we could have tested participants’ knowledge concerning the current treatment recommendations of acute hyponatraemia with a written questionnaire prior to the simulation course. However, we decided against this approach because we did not want to give participants a clue about which emergency scenario to expect.

Although a team of experienced simulation instructors developed the post-simulation survey, it did not undergo validation testing. In addition, the small sample size precluded statistical analysis for the majority of questions. Nonetheless, we believe that the questions were context-relevant and were suited to elicit the perception of participants on the usefulness and clinical relevance of the CA.

## Conclusions

The cognitive aid improved the implementation of evidence-based practices in a simulated intraoperative scenario. Cognitive aids with current medical content could help to close the translational gap between guideline publication and implementation in acute patient care. It is important that the cognitive aid should be familiar, in a format that has been used in practice and training.

## Additional file

Additional file 1: Text of cognitive aid (translated from German, not original formatting). The file contains the translated text of the original German version of the CA on “TURP syndrome”. (DOCX 18 kb)

## Abbreviations

AHF: Acute heart failure
CA: Cognitive aid
HTML5: Hyper text markup language version 5
ICU: Intensive care unit
LAST: Local anaesthetic systemic toxicity
ODS: Osmotic demyelinisation syndrome
PEEP: Positive end expiratory pressure
SBP: Systolic blood pressure
TURP: Transurethral resection of the prostate
