# Supplementary material for: Does an electronic cognitive aid have an effect on the management of severe gynaecological TURP syndrome? A prospective, randomised simulation study
Source: BMC Anesthesiol. 2017 May 30;17:72. doi: 10.1186/s12871-017-0365-8 (PMC5450103; doi:10.1186/s12871-017-0365-8)
Supplement: Additional file 1: — Text of cognitive aid (translated from German, not original formatting). The file contains the translated text of the original German version of the CA on “TURP syndrome”. (DOCX 18 kb) [file 12871_2017_365_MOESM1_ESM.docx]

**Additional file 1:** Text of cognitive aid (translated from German, not original formatting)

**TURP Syndrome**

| Hypervolaemia, hyponatraemia, and dilution coagulopathy resulting from resorption of large amount of hypotonic irrigation fluid |
| --- |

| **Highest priorities: Stop further fluid resorption,**  **correct hyponatraemia rapidly**  **START..--.**  _1_ **Call for help**  _2_  **Inform surgeon**  **AIRWAY – Spontaneously breathing patient..--.**  _3_  **Increase F_i_O_2_**   - (SpO_2_ > 94%)   _4_ Consider **early intubation** and controlled ventilation  **AIRWAY – Intubated patient P .--.**  _5_ **Increase F_i_O_2_  and PEEP** in response to ABG   - (SpO_2_ > 94%, P_a_O_2_>80 mmHg)   **Volume overload – Acute heart failure-**  _6_ Consider **decreasing preload: Nitroglycerine** infusion  _7_ Consider **increasing contractility: Dobutamine** infusion  _8_ Consider **increasing afterload:**  **Norepinephrine** infusion   - Optimise coronary perfusion   _9_ **Induce diuresis: Furosemide IV**  **Correction of osmolarity P .--.**  _10_ **Substitute** sodium chloride with **3% hypertonic saline**  **Laboratory analysis..--.**  _11_ **Continuously monitor ABG**  _12_ **Consider thrombelastography** | \| **DRUG DOSES and treatments** \| \| --- \| \| **Nitroglycerin infusion: 50mg**/50ml; rate 1–3 ml/h  **Dobutamine infusion:** **250mg**/50ml; rate 3–8 ml/h  **Norepinephrine infusion:** **5mg**/50 ml  **Furosemide**: 10–20 mg i.v. \|  \| **Treatment of HYPONATRAEMIA** \| \| --- \| \| **Acute (symptomatic) hyponatraemia should be rapidly corrected**  **Severe Hyponatraemia**   - Prompt infusion of **3% hypertonic saline** (**150 ml/20 min**) - Repeat infusion until a target of **5 mmol/l increase** in serum sodium concentration is achieved or until **neurologic symptoms improve**   **3% hypertonic saline (250ml)**   - **1ml/kg** increases serum sodium concentration by **1 mmol/l** - **1 bag (250ml)** increases serum sodium concentration by **3 mmol/l** - Pre-prepared **250-ml bags of 3% hypertonic saline** are stored in the **ICU** (phone 45200) \| |
| --- | --- | --- | --- | --- | --- |
